# Integrated energy system optimal scheduling considering the comprehensive and flexible operation mode of pumping storage

**DOI:** 10.1371/journal.pone.0275514

**Published:** 2022-10-05

**Authors:** Xinglin Yang, Jiaqi Chang, Zongnan Zhang, Jiaqi Zhang, Guanzhong Xu

**Affiliations:** School of Energy and Power, Jiangsu University of Science and Technology, Zhenjiang, Jiangsu, China; J.C. Bose University of Science and Technology, YMCA, INDIA, INDIA

## Abstract

The integrated energy system (IES) optimal scheduling under the comprehensive flexible operation mode of pumping storage is considered. This system is conducive to the promotion of the accommodation of wind and solar energy and can meet the water, electricity and heat needs of coastal areas far away from the energy center. In this study, the joint dispatch between double pumped storage power stations is used to accommodate wind and solar energy better and smooth their fluctuations on the grid. Through the scheduling of water between the high and low reservoirs of double pumped storage power stations and the reservoir of the seawater desalination plant, the impact of storage capacity constraints on pumped storage power plants is reduced. Moreover, the objective function to build the IES optimization scheduling model is to achieve minimum economic cost. The results reveal that the integrated energy system accommodates all wind energy and solar energy. As such, the system increases the average working time of the pumped storage unit by 1.9 hours and reduces the economic cost by 31.50%. Based on the simulation results, the model can enhance the accommodation capability of wind energy and solar energy and improve the system economy.

## 1 Introduction

### 1.1 Literature review

Under the double background of the energy and environment issues of the 21st century, integrated energy system came into being. This system facilitates the integration of natural gas, electric energy, heat energy, wind energy, solar energy, and other kinds of energy in a certain region and coordinates the planning and optimization of the generation, conversion, storage, transmission, distribution, and other processes of all kinds of energy to improve the energy utilization rate [1]. At present, wind and solar energy are among the hotspots of new energy application and research [2], and their share in the comprehensive energy system increases year by year. These two energy sources are a great burden to the power system because of their large randomness and intermittency, which result in more wind and solar energy curtailment. To accommodate wind energy and solar energy more efficiently and reduce their curtailment, energy storage devices often store them during low load periods and release them during peak load periods to bear the electrical load [3–5]. Pumped storage is one of the important solutions to these issues [6]. Hydropower storage is currently the most mature technology and the largest energy storage technology for equipment. This widely used mechanism has the characteristics of flexible start and stop and flexible function switching; moreover, it can smoothen the fluctuation of wind and solar energy generation better [7, 8]. Muhammad et al. [9] conducted economic analysis and optimization on the combination of various renewable energy sources (solar and wind) and storage technologies (battery, pumped hydro storage, and hybrid storage) in the off-grid power supply system. The results show that the hybrid pumped battery storage can ensure 100% power supply with minimum cost and reduce energy curtailment. Ma et al. [10] proposed a system design optimization scheme for a proposed hybrid solar–wind-pumped storage system. The results show that wind turbines can increase economy and reduce the size of energy storage. Xu et al. [11] designed a combined operation system of wind, solar, and pumping storage for remote area. The purpose is to pursue the economy of system operation on the basis of accommodating wind and solar energy to ensure stable power supply. Ma et al. [12] established a two-layer stochastic optimization model with wind storage, and the system benefits are optimized by Nash negotiation and Shapley value method. Makhdoomi et al. [13] used a modified crow search algorithm to optimize a hybrid energy system consisting of a photovoltaic diesel generator and pumped hydro storage with the lowest fuel consumption as the objective function. Rathore et al. [14] uses analytical technique and Monte Carlo simulation to evaluate the reliability of the photovoltaic-wind energy system incorporated with a pumped storage hydro plant and compared the evaluation results of the two methods. Studies demonstrate that the pumped storage-based energy storage system is more reliable and environmentally friendly than the battery storage system.

In this article, the fresh water in coastal areas mainly comes from the desalination system, and the higher power consumption of the system is a greater burden for areas far from the grid [15]. For the above problems, Marco et al. [16] examined the synergistic effect of seawater desalination and photovoltaic power generation to increase the sustainability of the system, and the optimal capacity combination method of microgrid components is explored with the goal of minimizing the total cost of the microgrid. Luca et al. [17] studied a single-effect thermal water generation unit coupled with CSP. The unit can produce 60–75 L of fresh water per day. Liu et al. [18] used mixed-integer linear programming algorithm to plan the optimal capacity of pumped storage-seawater desalination system for renewable energy sources with maximum economic benefit as the objective function. Ghaithan et al. [19] proposed using a grid-connected hybrid solar-wind system to power a small-scale Reverse Osmosis (RO) desalination unit and analyzed its performance in the weather conditions of Saudi Arabia’s eastern province.

### 1.2 Research gap and motivation

Various scholars [9–14] have conducted relatively sufficient research on the aspects of accommodating renewable energy and improving the system economy of pumped storage power. However, they have not considered the problem that the reservoirs required for pumped storage power plants are affected by the special terrain and cannot be further promoted. The higher requirements on the terrain restrict the capacity of the high-level reservoir of the pumped storage power station, and the smaller reservoir capacity results in a limited capacity for wind and solar energy. In addition, to reduce the fluctuation caused by renewable energy to the power grid, this study does not consider the integration of wind energy and solar energy into the power grid or directly participate in the supply of user load. However, the pumped storage power station absorbs the energy and then participates in the system energy dispatching. Under this setting, wind energy and solar energy can only be stored in the pumped storage power station and then used.

However, the pumped storage power station can only store or discharge energy at the same time. At the discharge time (affected by the storage capacity constraint or the power supply demand of the system), given the lack of storage equipment for wind energy and solar energy, a large amount of energy is wasted. Notably, aforementioned studies have not discussed such issues extensively.

Various problems also persist in the storage and transportation of fresh water resources in [16–19]. To deliver fresh water to target users better, fresh water reservoirs can be placed at a higher altitude relative to the target user. In this way, the height difference of the terrain can be used in exchange for higher kinetic energy, thereby reducing the complexity of the downstream user’s conveying system.

In response to the above problems, this article takes the coastal areas far away from the energy center as the object and aims at the water, electricity, and heat problems in this area. Then, it constructs an IES considering the comprehensive and flexible operation mode of pumping storage.

### 1.3 Contribution

The main innovations and contributions are as follows:

- This study adopts the form of joint operation of two small pumped storage power stations of different scales to solve the problem in which the capacity of a single pumped storage power station is difficult to expand due to high terrain requirements.
- By adopting the mode of joint operation of two pumped storage power stations, one pumped storage power station can be in the discharge state, while the other can be in the charge state (accommodate wind energy and solar energy). This mode is expected to solve the waste of wind energy and solar energy of the single pumped storage power station mentioned above.
- In order to reduce the influence of storage capacity constraints on the operation of pumped storage power station under the premise of ensuring the fresh water transportation capacity. This study proposes to solve this problem through the system water scheduling. For details, see Section 2.2.
- This study takes the minimum economic cost as the objective function to construct an IES optimal scheduling model. Typical daily data in a certain area are selected, and the improved gray wolf algorithm is used for simulation. The results are analyzed, after which the theory is verified.

### 1.4 Paper organization

The first section is the introduction. This portion briefly describes the research background and research status, analyzes the problems of pumped storage power stations in accommodating renewable energy, such as wind energy and solar energy, and the problems of seawater desalination devices in energy consumption and fresh water transportation, and provides the contribution and organization of the paper.

The second section includes the system description and mathematical modeling. The components of the system are introduced, and the corresponding mathematical models are established.

The third section is the economic dispatch model of the integrated energy system. The working principle and improvement process of the gray wolf algorithm are introduced, and the solution process of the algorithm to the economic dispatch model is explained.

The fifth section is the conclusion. The discussion in the previous sections are summarized, and some prospects for the future are subsequently proposed.

## 2 System description and mathematical modeling

### 2.1 Wind-solar-pumped storage energy supply system

**Fig 1:**
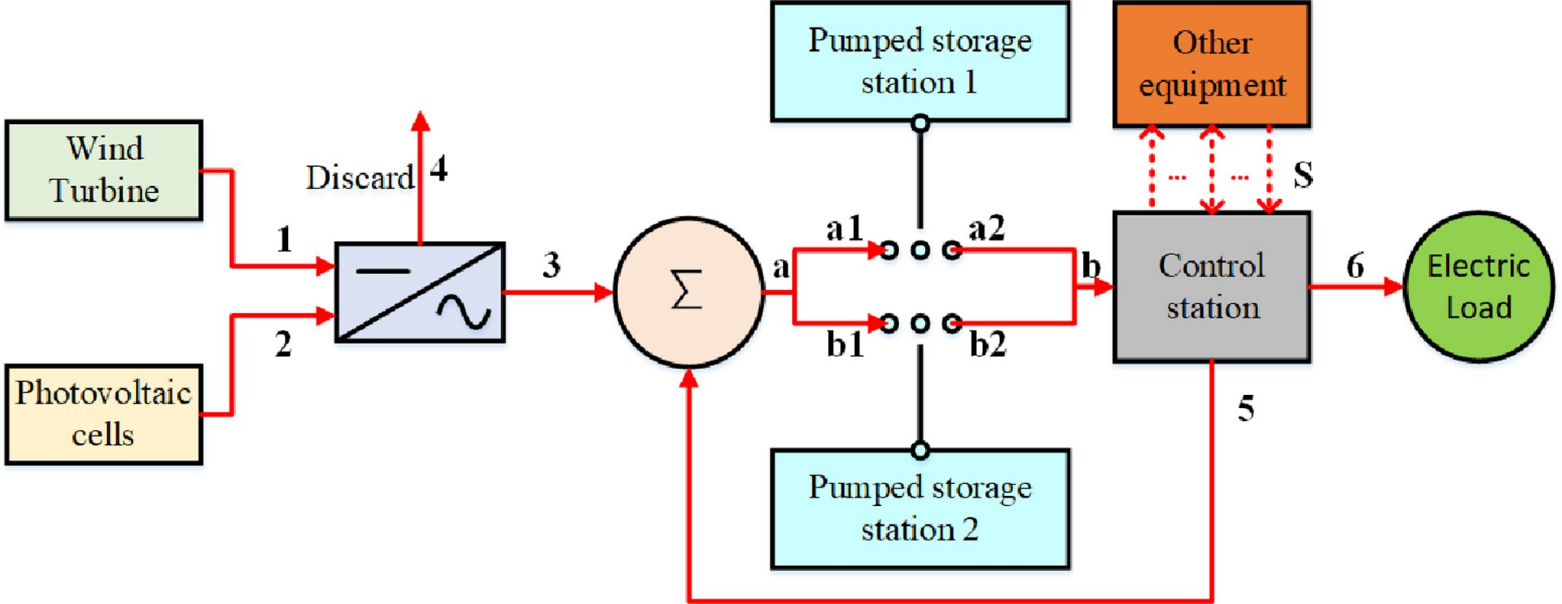
Wind-solar-pump storage energy supply system.

Wind energy and solar energy are volatile and random. When they are connected to the power grid, the phenomenon of abandoning wind and solar energy occurs. In this research, renewable energy is stored in the energy storage system and released according to the system scheduling requirements, thereby improving the utilization rate of wind and solar energy. Based on the above concepts, a wind-solar-pump storage energy supply system (WSPS) is established, as shown in **Fig 1**.

As shown in **Fig 1**, first, wind energy and solar energy are converted by inverters (1, 2–3). Then, they are stored by the pumped storage power station, and the unstored energy is discarded (4). The relevant relationship is:

$$P_{W}^{e}\left(t\right)+P_{V}^{e}\left(t\right)=P_{WV,dis}\left(t\right)+e_{3}\left(t\right).$$ (1)

where, $P_{W}^{e}\left(t\right)$ and $P_{V}^{e}\left(t\right)$ are the output power of the wind turbine and photovoltaic cells in period t, respectively; *P*_*WV*, *dis*_(*t*) is for unused wind energy and solar energy at time t; *e*_*i*_ is the electric power of energy flow i.

The pumped storage power station has three working states: pumped storage, power generation, and shutdown. The control station sets the working states of the pumped storage power station 1 and 2 according to the system requirements. When the pumped storage power stations 1 and 2 are in the pumped storage state, the control station will supplement the electric power (5) if the electric power generated by wind energy and solar energy (a) is less than the pumped water power. When pumped storage power stations 1 and 2 are in a discharge state, the released electric energy (b) will flow to the control station. The relevant relationship is:

$$e_{3}\left(t\right)+e_{5}\left(t\right)=e_{a}\left(t\right)$$ (2)

$$\left\{\begin{array}{c} P_{ps1}^{c}\left(t\right)+P_{ps2}^{c}\left(t\right)=e_{a}\left(t\right)\\ P_{ps1}^{d}\left(t\right)+P_{ps2}^{d}\left(t\right)=e_{b}\left(t\right)\\ I_{psi}^{c}\left(t\right)P_{psi}^{c,\text{min}}⩽P_{psi}^{c}\left(t\right)⩽I_{psi}^{c}\left(t\right)P_{psi}^{c,\text{max}}\\ I_{psi}^{d}\left(t\right)P_{psi}^{d,\text{min}}⩽P_{psi}^{d}\left(t\right)⩽I_{psi}^{d}\left(t\right)P_{psi}^{d,\text{max}}\\ I_{psi}^{c}/I_{psi}^{d}\in \left\{0,1\right\}\\ I_{psi}^{c}\left(t\right)+I_{psi}^{d}\left(t\right)⩽1 \end{array}\right.$$ (3)

where, $I_{psi}^{c}\left(t\right)$/$I_{psi}^{d}\left(t\right)$ is the status flag bit of the pumped storage/discharge of pumped storage power station i in period t; $P_{psi}^{c}\left(t\right)$/$P_{psi}^{d}\left(t\right)$ is the pumping/discharging power of pumped storage power station i in period t; $P_{psi}^{c,\text{max}}$ is the upper and lower limit of the pumping power of the pumped storage power station i; $P_{psi}^{d,\text{min}}$/$P_{psi}^{d,\text{max}}$ is the upper and lower limit of the discharge power of the pumped storage power station i.

The control station is responsible for controlling the flow direction and size of each energy flow and maintaining the system power balance under the premise of satisfying the user’s load demand. The relevant relationship is:

$$e_{b}\left(t\right)-e_{5}\left(t\right)+e_{s}\left(t\right)+e_{6}\left(t\right)=0$$ (4)

Under this comprehensive and flexible operation mode, the working states of pumped storage power stations 1 and 2 are independent of each other and are regulated by the control station. For example, during the peak period of wind energy and electric energy, the control station can change the state of both pumped storage power stations into a pumped-storage state to improve the power stations’ ability to absorb wind and solar energy. During the trough period of wind and electric energy, the control station can make turn one pumped storage power station into a pumped-storage state for accommodating wind energy and solar energy, and the other pumped storage power station can be in a power-generating state.

### 2.2 System water scheduling

**Fig 2:**
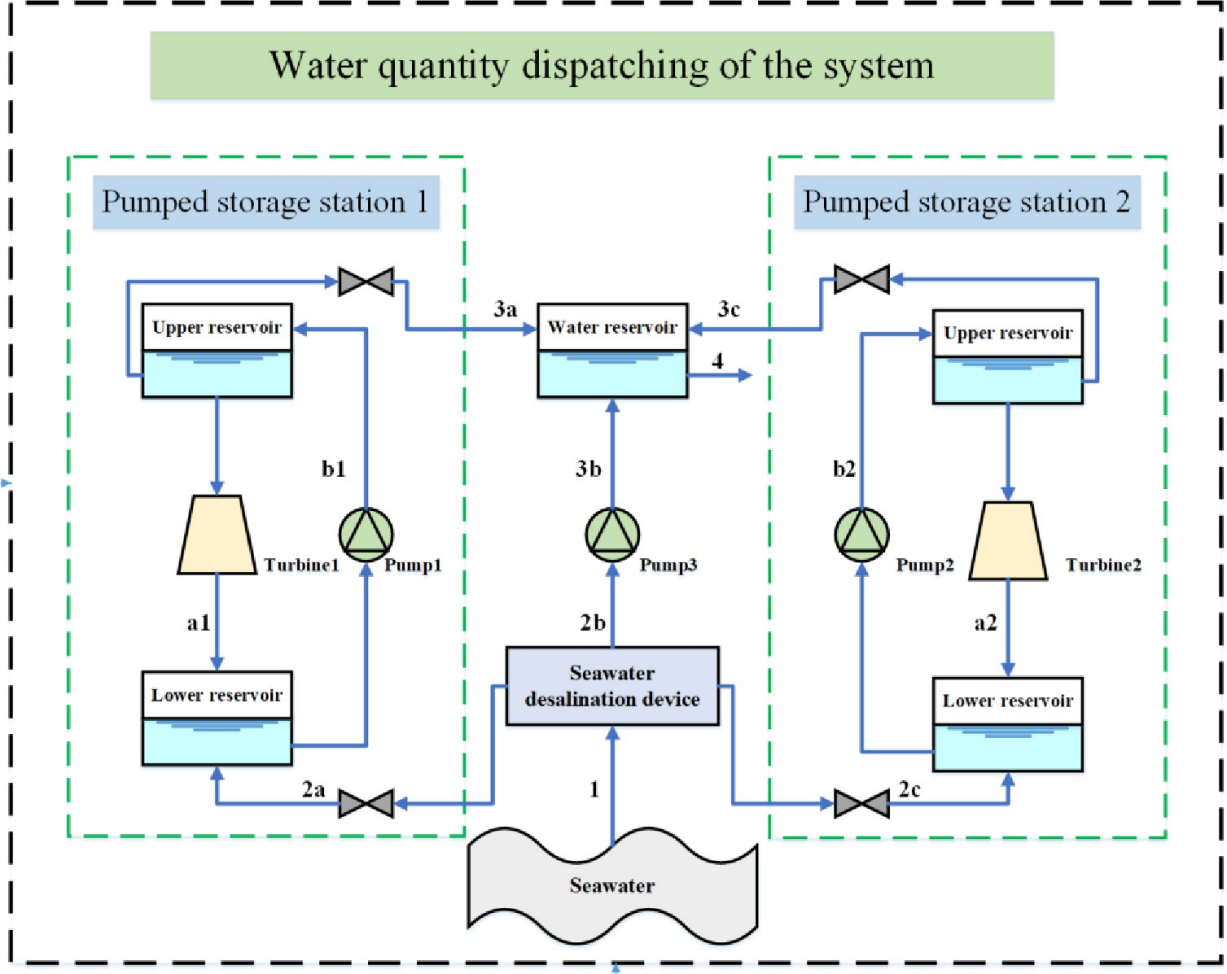
Framework of system water scheduling.

A pumped storage power station is a hydropower station that uses electric energy when the power load is too low to pump water to a high-level reservoir. This station discharges water to a low-level reservoir to generate electricity during the peak power load period. The upper and lower limits of the storage capacity of the high/low reservoirs of pumped storage power stations affect the pumping and discharge power. In this study, this limitation is reduced by the water scheduling of the high/low level reservoirs of the double pumped storage power station and the reservoir of the seawater desalination device, as shown in **Fig 2**.

As shown in **Fig 2**, system water scheduling is divided into water scheduling 1 and water scheduling 2. Water scheduling 1 is water scheduling between the high and low reservoirs of the pumped storage power station. Water scheduling 2 is water scheduling between the reservoir of seawater desalination plant and reservoirs of pumped storage power stations.

Water scheduling 1: When the pumped storage power station is pumped and stored, the high-level reservoir flows to the low-level reservoir (a1/b1) to drive the turbine to do the work. When the pumped storage power station discharges, the low-level reservoir is transported to the high-level reservoir (a2/b2) by the water pump.

Water scheduling 2: First, the downstream seawater is converted into fresh water (1) by a seawater desalination device (located at the downstream). Second, the fresh water can flow to the low-level reservoir (2a / 2c) to increase the water volume of the low-level reservoir, and the remaining fresh water is pumped to the reservoir of seawater desalination plant (3b). Finally, fresh water from the reservoir of seawater desalination plant flows to the user group (4), which can be replenished by the high reservoir of the pumped storage power station (3a / 3c) when it is insufficient. The correlation is as follows:

$$\left\{ \begin{array}{c} m_{2a}\left(t\right)=m_{3a}\left(t\right)\\ m_{4}\left(t\right)=m_{users}\left(t\right) \end{array}\right.$$ (5)

where, *m*_*i*_ (*t*) is the flow rate of water flow i in period t; *m*_*users*_ (*t*) is the user’s fresh water demand in period t.

### 2.3 Multi-energy complementary system

**Fig 3:**
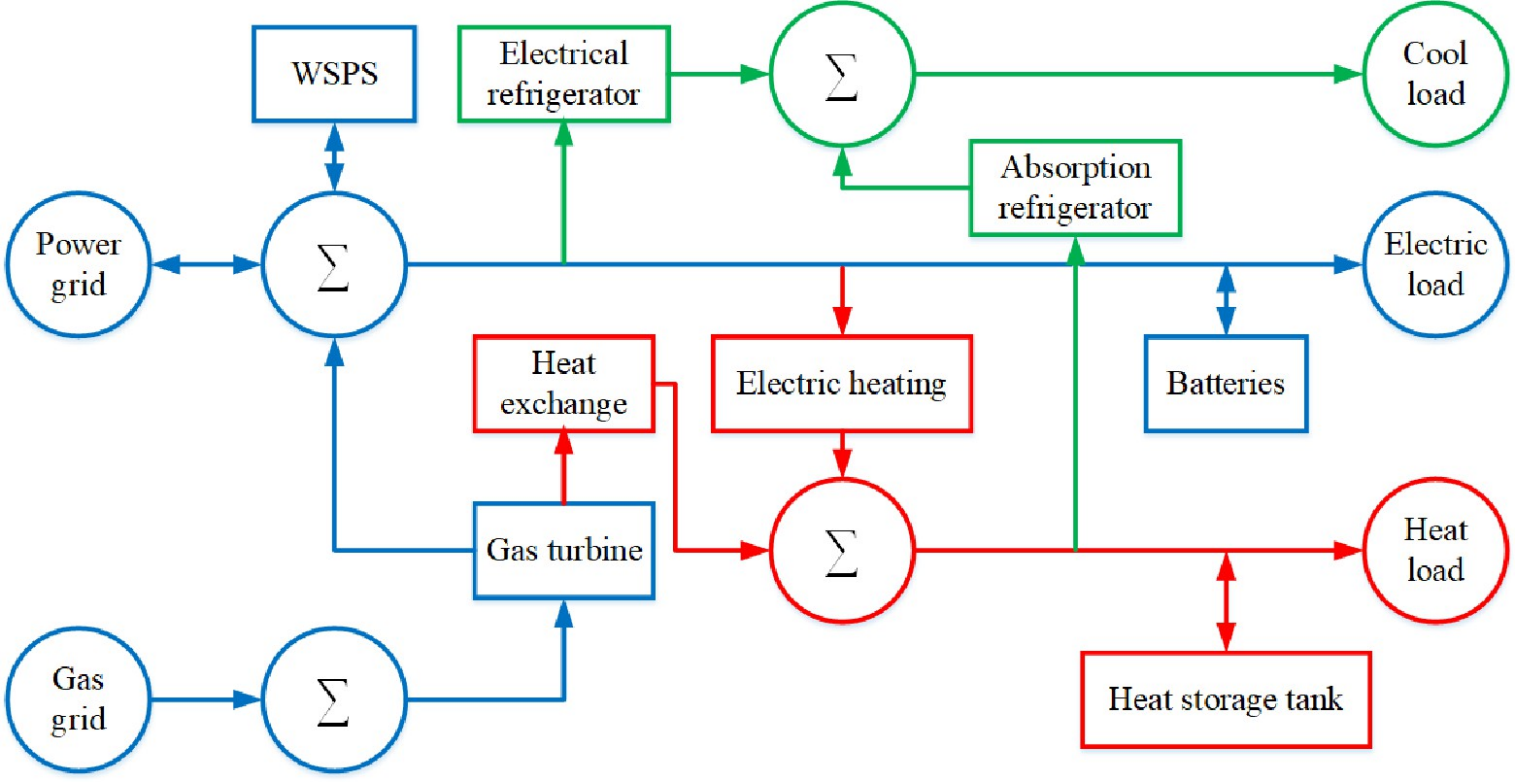
Multi-energy complementary system.

In this study, users have water demand, electricity demand, cooling demand, and heat demand, among which the water demand is borne by the seawater desalination device, which will not be discussed in this section. To meet the user’s cooling, heating, and electric load requirements, a multi-energy complementary system is established in this work [20–23], as shown in **Fig 3**.

**Fig 3** demonstrates that WSPS, gas turbines, batteries, and large power grids are used to bear the electrical load. Meanwhile, waste heat boilers, electric heating, and heat storage tanks are used to bear the thermal load. Absorption chillers and distributed air conditioners are used to bear the cooling load. The relevant equality constraints and inequality constraints are as follows.

Power balance:

$$E\left(t\right)=e_{b}\left(t\right)-e_{5}\left(t\right)+P_{MT}^{e}\left(t\right)+P_{grid}^{e}\left(t\right)-P_{ES}^{c}\left(t\right)+P_{ES}^{d}\left(t\right)-P_{EB}^{e}\left(t\right)-P_{EC}^{e}\left(t\right)+P_{DES}^{e}\left(t\right)$$ (6)

where, $P_{MT}^{e}\left(t\right)$ is the power generated by the gas turbine in period t; $P_{grid}^{e}\left(t\right)$ is the electricity purchased by the large power grid in period t; $P_{ES}^{c}\left(t\right)$/$P_{ES}^{d}\left(t\right)$ is the charging/discharging power of the battery in period t; $P_{EB}^{e}\left(t\right)$/$P_{EC}^{e}\left(t\right)$ is the power consumption of electric refrigeration/electric boilers in period t; *E*(*t*) is the electrical load demand of the user in period t; $P_{DES}^{e}\left(t\right)$ is the power consumption of desalinated seawater in period *t*.

Heat balance:

$$H\left(t\right)=P_{RB}^{h}\left(t\right)+P_{EB}^{h}\left(t\right)-P_{HST}^{c}\left(t\right)+P_{HST}^{d}\left(t\right)-P_{AC}^{h}\left(t\right)$$ (7)

where, $P_{RB}^{h}\left(t\right)$/$P_{EB}^{h}\left(t\right)$ is the output thermal power of the waste heat boiler/heating electric boiler in period t; $P_{HST}^{c}\left(t\right)$/$P_{HST}^{d}\left(t\right)$ is the charge/discharge power of the heat storage tank in period t; $P_{AC}^{h}\left(t\right)$ is the input thermal power of the absorption chiller in period t; *H*(*t*) is the heat load demand of the user in period t.

Cold balance:

$$Co\left(t\right)=P_{AC}^{c}\left(t\right)+P_{EC}^{c}\left(t\right)$$ (8)

where, $P_{AC}^{c}\left(t\right)$/$P_{EC}^{c}\left(t\right)$ is the output cooling power of the absorption refrigerator/electric refrigerator in period t; *Co*(*t*) is the cooling load demand of the user at period t.

Mathematical models of each micro-source component in the system [24–26] are presented in S1 Appendix.

## 3 Economic dispatching model of integrated energy system

The IES optimal scheduling model established in this study is a single objective model, and the objective is to minimize the sum of operation cost, maintenance cost, and environmental cost. Constraints, such as the upper and lower limits of the heat generation power of waste heat boiler, have been described above and will not be repeated here. Details are presented in S2 Appendix.

### 3.1 Solution of the algorithm

#### 3.1.1 Introduction and improvement of the algorithm

The optimization of the IES is a non-linear optimization problem with complex constraints and high solving dimensions. This study uses the improved gray wolf algorithm to solve the problem [27, 28].

Gray Wolf Optimizer (GWO) is a new intelligent algorithm proposed by Mirialili et al. in 2014 [29]. The optimization process of GWO involves the steps of hierarchy, tracking, surrounding, and attacking the prey. Kadali et al. [30] used the gray wolf optimization algorithm to optimize the scheduling of thermal power systems. Zheng et al. [31] used the gray wolf optimization algorithm to balance the load among smart microgrids, user demand, and service providers. Shilaja et al. [32] proposed a hybrid algorithm based on the enhanced grey wolf optimization and dragonfly algorithm to deal with the optimal power flow problem. Although the general GWO has better performance than most intelligent algorithms, it is not suitable for handling functions with high complexity. How to improve the balance between global search and local convergence is one of the important directions to improve the performance of the GWO. In this study, some improvements have been made in combination with the particle swarm algorithm [33], the Harris Hawk algorithm [34], and the bat algorithm [35]. The specific improvement methods are shown in S3 Appendix.

#### 3.1.2 Solving steps of the algorithm

The improved gray wolf algorithm is applied to the optimization solution steps of the integrated energy system scheduling as follows:

**Step 1:** Set the parameters and data of the IES optimization model.
**Step 2:** Set the parameters of the algorithm and initialize the gray wolf population.
**Step 3:** Use Monte Carlo to simulate the continuous exploration process of the IES scheduling scheme, and select a better scheduling scheme as the initial solution set of the optimization algorithm, thereby updating the entire gray wolf population.
**Step 4:** Use FCM clustering algorithm to group gray wolf populations (IES scheduling scheme) to prepare for the cooperative hunting process of wolves.
**Step 5:** Select the first wolf in each group of wolves, that is, the optimal plan in each group of scheduling plans, and use the displacement update formula in the particle swarm algorithm to update the position of each wolf.
**Step 6:** Continuously update the escape position of the prey (IES global optimal scheduling scheme) using the Harris Hawks Optimizer (HHO), and use Monte Carlo random simulation to find the best escape position of the prey.
**Step 7:** Randomly extract wolves from each group except wolves with probability P, that is, select some of the schemes in each group except the optimal one, and update their positions with the displacement update formulas of the grey wolf algorithm and particle swarm algorithm.
**Step 8:** Randomly extract the remaining wolves except the head wolf with probability (1-P), and update their positions with the displacement update formula of the bat algorithm.
**Step 9:** Add a memory library to store the location of wolves in each group during the previous n iterations, and select the individual with higher crowding as the current individual through the crowding analysis. Save the optimal individual among wolves and the IES global optimal scheduling scheme.
**Step 10:** Determine whether the maximum number of iterations has been reached, output the optimal individual if it is satisfied, and jump to step 5 instead until the termination condition is met.

## 4 Analysis of examples

In this paper, the basic data of a certain area and the actual project are used to construct calculation examples, and the effectiveness of the research is demonstrated through simulation analysis. The scheduling period of the calculation example is 24h, and the length of a single period is 1h.

### 4.1 Basic data

**Fig 4:**
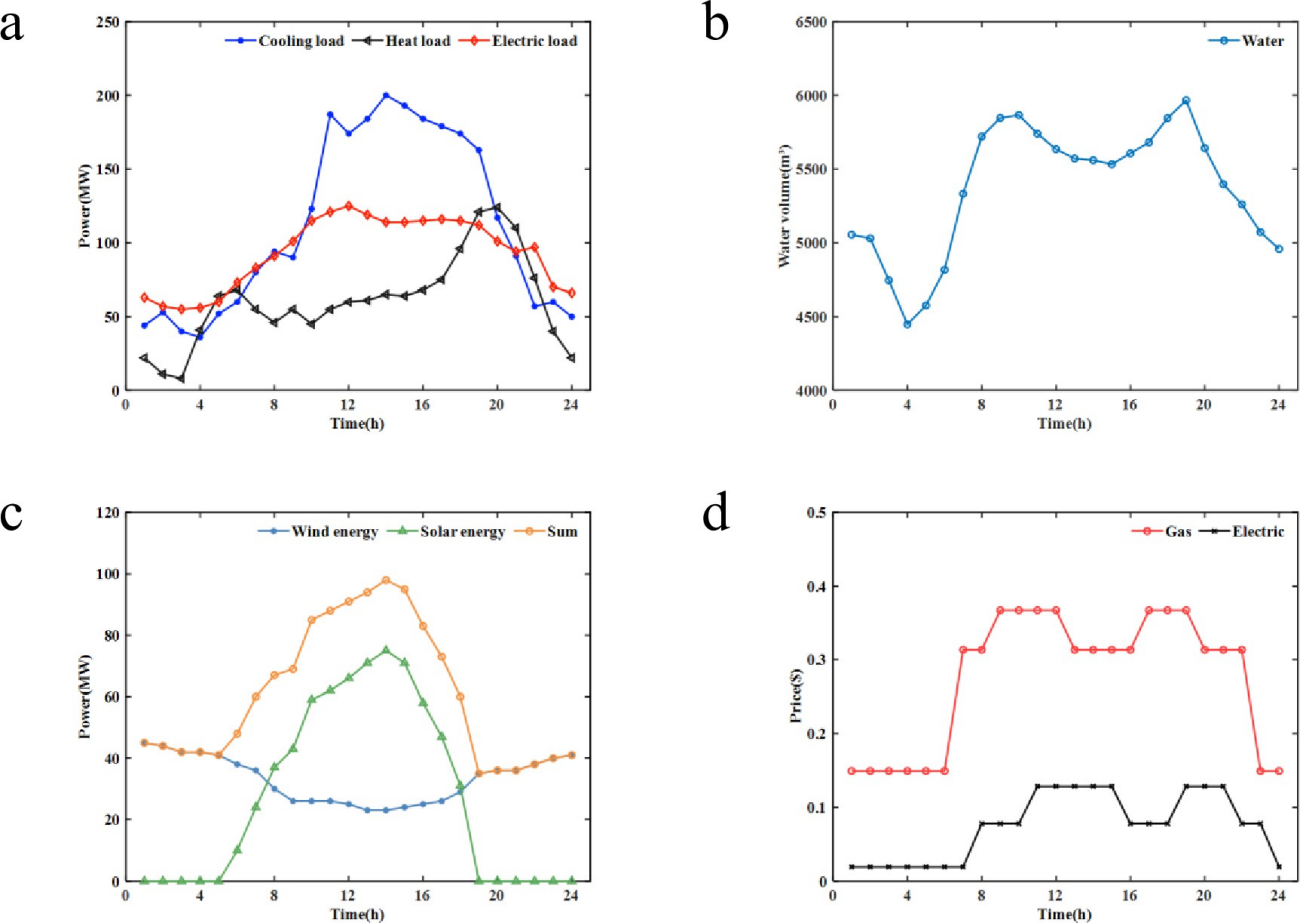
Load demand for the area: (a) Hourly electrical, heating and cooling loads data, (b) The fluctuation in the demand for desalinated water in a 24-hour period, (c) The time-varying curve of wind energy and solar energy, (d) Electricity price ($/kW·h) and gas price ($/m3).

In this study, the parameters of the pumped storage power station in [36], hourly electrical, heating, and cooling loads data in [37], the fluctuation in the demand for desalinated water in a 24-hour period [18], and the time-varying curve of wind energy, solar energy in [38] are referred for model construction and algorithm solution. Among them, pumped storage unit related data are presented in S1 Table, while the reservoir data of the pumped storage power station are shown in S2 Table and Fig 4A is the forecast curves of the heat and cooling load, electrical load, and cooling load; Fig 4B is the forecast curves of the demand for desalinated water; Fig 4C presents the forecasts for wind and photovoltaic power generation.

Refer to micro-source equipment parameters and energy prices in [24, 36, 39–44] to make the following settings. The maximum output power and minimum output power of the gas turbine are 114 MW and 28.5 MW, respectively. The upper and lower limits of battery operating power are 40 MW and -40 MW, and the initial and maximum capacities are 160 MW·h and 480 MW·h, respectively. The upper and lower limits of the operating power of the heat storage tank are 200 MW and -200 MW, respectively, and the initial and maximum capacities are 100 MW·h and 400 MW·h, respectively. The rated power of electric heating, electric refrigerator, waste heat boiler, and absorption refrigerator are 120 MW, 150 MW, 150 MW, and 150 MW, respectively. The time-sharing purchase electricity price and gas price are shown in Fig 4D [40]. Micro-source equipment correlation coefficient are shown in S3 Table [24, 41]. Prices for the maintenance of related equipment are shown in S4 Table [24, 41]. Carbon tax price is set to 20 $/t [42]. Gas turbine CO_2_ emission is 724 kg/MW·h [43]. The cost of the desalination unit is set to 0.857 $/m^3^ [44]. The pumping power of the pump is 130 MW. The pumping flow is 37.4 m^3^/MW·h [36].

To demonstrate the effectiveness of the proposed model, this article sets up four cases based on the actual situation, as follows:

Case 1: IES economic dispatch without considering pumped storage power station
Case 2: Based on Case 1, consider pumped storage power station 1
Case 3: Based on Case 2, consider the water scheduling between the reservoir of seawater desalination plant and reservoirs of pumped storage power station 1 (water optimal dispatch 1)
Case 4: Based on Case 3, consider the joint dispatch between pumped storage power stations 1 and 2, and the water scheduling between the reservoir of seawater desalination plant and reservoirs of pumped storage power station 1 and 2 (water optimal dispatch 2)

Note: Pumped storage power station 1 is equipped with pumped storage unit PU-1 and PU-2. The maximum generating power is 90 MW, the pumping power is 80.2 MW, and the configuration reservoir U-1 is set. Pumped storage power station 2 install pumped storage unit PU-1. The maximum power generation is 60 MW, pumping power is 40.1 MW, and configuration reservoir U-2 is set. Among them, PU-1 and PU-2 are shown in **S1** Table, and U-1 and U-2 are shown in **S2** Table.

### 4.2 Optimization results and analysis

#### 4.2.1 Result and analysis of pumped storage unit combination

Table 1A–1C list the combination of pumped-storage units in each period of summer in Cases 2–4, in which units G1 and G3 are PU-1 and G2 is PU-2. For unit Gi, “0” means the unit is not working, “1” means the unit is generating electricity, and “-1” means the unit is pumped storage. In this study, pumped storage units are mainly used to accommodate wind and solar energy to reduce the rate of abandonment of wind and solar energy and to minimize the impact of wind and solar energy fluctuations on the grid.

**Table 1:** Unit combination of each time period in Cases 2–4.

**(a) Unit combination of each time period in Cases 2–4**																								
Unit	Hours 1–24																							
	1	2	3	4	5	6	7	8	9	10	11	12	13	14	15	16	17	18	19	20	21	22	23	24
G1	1	1	-1	-1	-1	-1	-1	0	1	-1	1	1	1	1	-1	-1	-1	1	1	1	1	1	0	0
G2	1	1	0	0	0	0	-1	1	1	-1	1	1	1	1	-1	-1	-1	1	1	1	1	1	-1	-1
**(b) Unit combination of each time period in Case 3**																								
Unit	Hours 1–24																							
	1	2	3	4	5	6	7	8	9	10	11	12	13	14	15	16	17	18	19	20	21	22	23	24
G1	-1	-1	-1	-1	-1	-1	-1	-1	-1	-1	-1	-1	-1	-1	-1	-1	-1	-1	-1	-1	-1	-1	-1	-1
G2	-1	-1	-1	-1	-1	-1	-1	-1	-1	-1	-1	-1	-1	-1	-1	-1	-1	-1	-1	-1	-1	-1	-1	-1
**(c) Unit combination of each time period in Case 4**																								
Unit	Hours 1–24																							
	1	2	3	4	5	6	7	8	9	10	11	12	13	14	15	16	17	18	19	20	21	22	23	24
G1	-1	-1	-1	-1	-1	-1	-1	-1	-1	-1	-1	-1	-1	-1	-1	-1	-1	-1	0	0	0	0	0	-1
G2	-1	-1	-1	-1	-1	-1	-1	-1	-1	-1	-1	-1	-1	-1	-1	-1	-1	-1	1	1	1	1	1	-1
G3	1	1	1	1	1	1	1	1	1	-1	-1	-1	-1	-1	-1	-1	1	1	-1	-1	-1	-1	-1	1

Comparing Case 3 and Case 2, in Case 2, the G1 discharge period is 12 hours, the energy storage period is 9 hours, and the shutdown period is 3 hours; the G2 discharge period is 13 hours, and the energy storage period is 7 hours. The results show that considering a single pumped storage power station can meet the work requirements of discharge and energy storage. However, the shutdown state has an average of 3.5 hours of work period. In Case 3, the time period during which each unit is in the working and pumped storage state is significantly increased, which shows that considering water optimal dispatch 1, the impact of storage capacity constraints on pumped storage power plants is reduced. Moreover, it is mainly used to accommodate wind and solar energy. In this case, the pumped-storage unit is overused for pumped storage, and its power generation function is ignored, thus resulting in a waste of the unit’s functions.

By comparing Cases 2, 3, and 4, in Case 4, G1 is in the energy storage period except for periods 19–23 when it is not working. G2 is in a discharge state in periods 19–23, and all other periods are in energy storage period. G3 discharge period is 12 hours, and the energy storage period is 12 hours. The results show that considering the joint dispatch of pumped storage power stations 1 and 2 and water optimal dispatch 2, only the average 1.6 hours of working time is in a shutdown state, which is 1.8 lower than that of case 2. Hence, the overall discharge/storage ratio of the pumped storage power station is significantly higher than that of Case 3. From what has been discussed above, the comprehensive index of this scheme has a strong balance.

#### 4.2 2 Analysis of wind and solar energy accommodation

**Fig 5:**
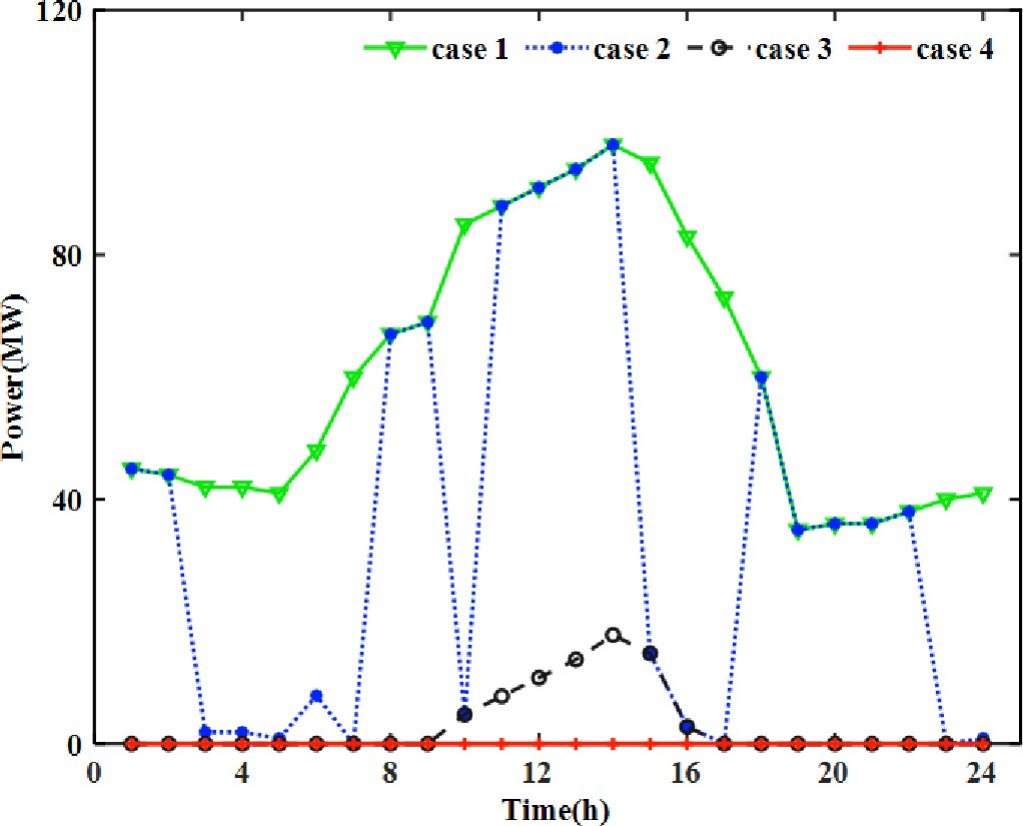
Curtailment of wind and solar energy by time period.

In this article, the scenario in which the wind energy and solar energy have not been accommodated by the pumped storage power station is wind and solar energy curtailment. The wind and solar energy curtailment for each period in Cases 1–4 is shown in Fig 5. In Case 1, wind and solar energy curtailment occurs in each period because no pumped storage power station is used. In Cases 2–4, the wind and solar energy curtailment continue to decrease, and in Case 4, the wind and solar energy curtailment is almost zero.

**Fig 6:**
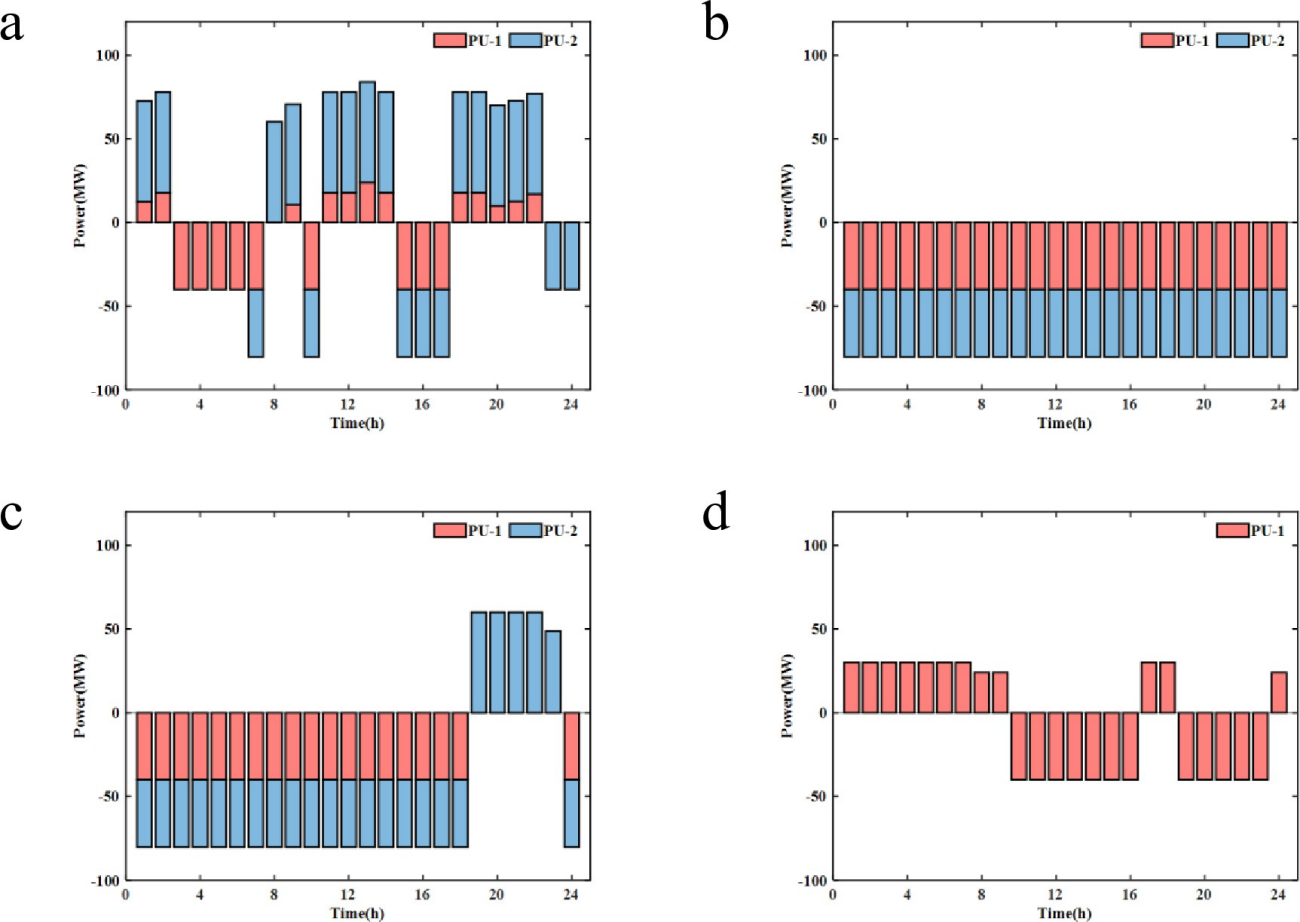
Output of pumped storage power station in Cases 2–4 (a) Output of pumped storage power station 1 in Case 2, (b) Output of pumped storage power station 1 in Case 3, (c) Output of pumped storage power station 1 in Case 4, (d) Output of pumped storage power station 2 in Case 4.

**Fig 7:**
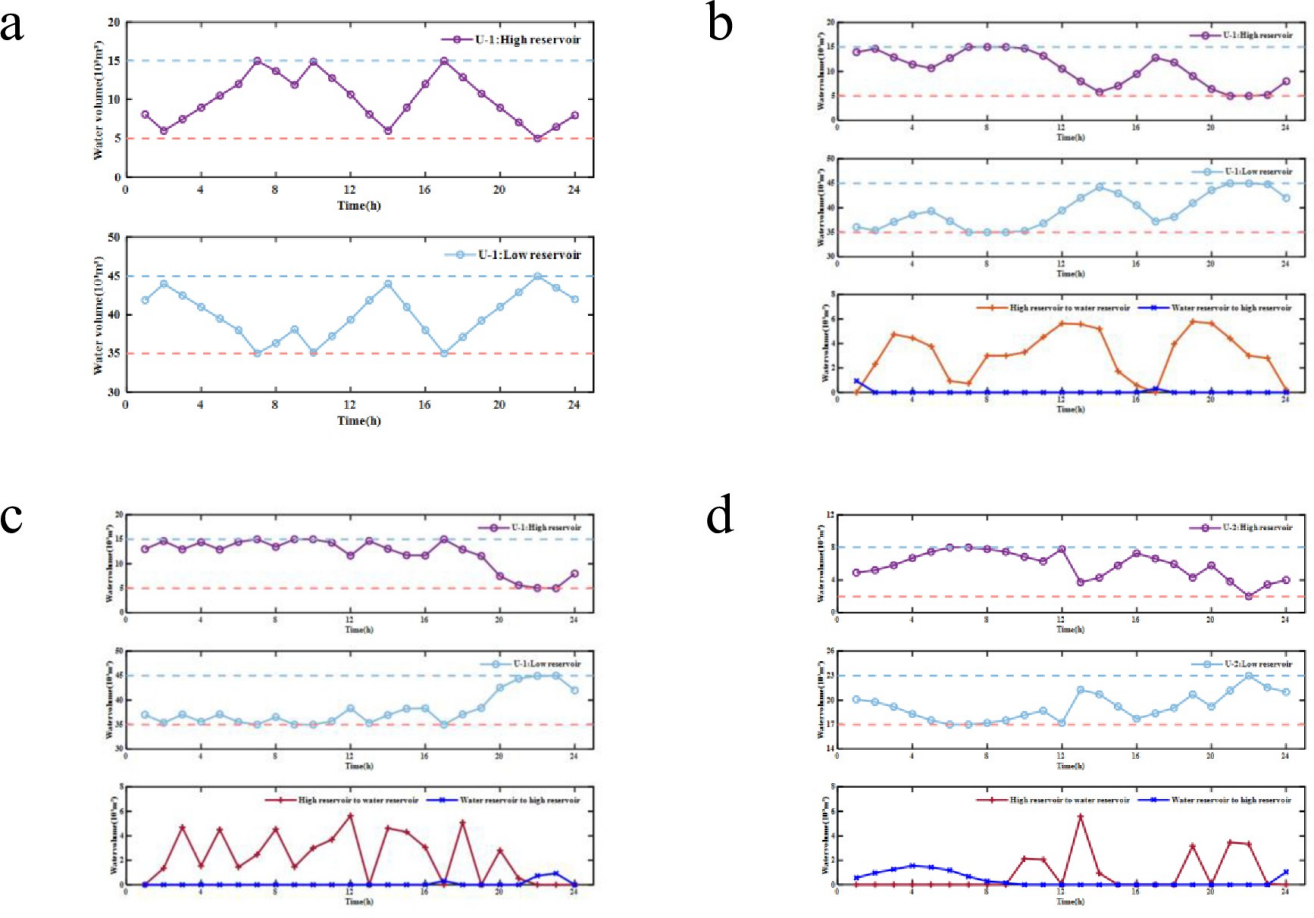
Variation of water volume in the reservoir in Case 2–4: (a) Variation of water volume of U-1 in Case 2, (b) Variation of water volume of U-1 in Case 3, (c) Variation of water volume of U-1 in Case 4, (d) Variation of water volume of U-2 in Case 4.

Comparing Case 2 and Case 1, adding pumped storage power station 1 can effectively accommodate wind and solar energy. However, in the period except 1–2, 8–9, 11–14, 18–22, etc., the pumped storage power station does not effectively accommodate wind and solar energy. According to Figs 6A and 7A, the pumped storage unit is in the state of generating electricity during these periods. The main reason is that the pumped storage power station can only be in the state of storing energy or generating electricity at the same time. Therefore, although adding a single pumped storage power station can accommodate wind and solar energy, the effect is limited.

Comparing Case 3 with Case 2, the wind and solar energy curtailment in Case 3 decreases significantly, thus indicating that increasing water optimal dispatch 1 can effectively reduce the impact of storage constraints and better accommodate wind and solar energy. However, as shown in Figs 6B and 7B, the pumped storage power station will be in the pumped storage state during the whole period of time, and its power generation function has not been applied, thus resulting in a waste of system resources.

Comparing Case 4 and Case 3, the wind and solar energy curtailment in Case 4 is zero. It shows that considering the joint dispatch of pumped storage power stations 1 and 2 and water optimal dispatch 2 can further accommodate wind and solar energy. In addition, as shown in Figs 6C and 6D and 7C and 7D, the proportion of the power generation period and the energy storage period of pumped storage power stations 1 and 2 is higher than that of Case 3 but lower than that of Case 2.

After the above analysis, Case 4 has had a good impact on improving the economic dispatch of the integrated energy system.

#### 4.2 3 Result and analysis of equipment output

**Fig 8:**
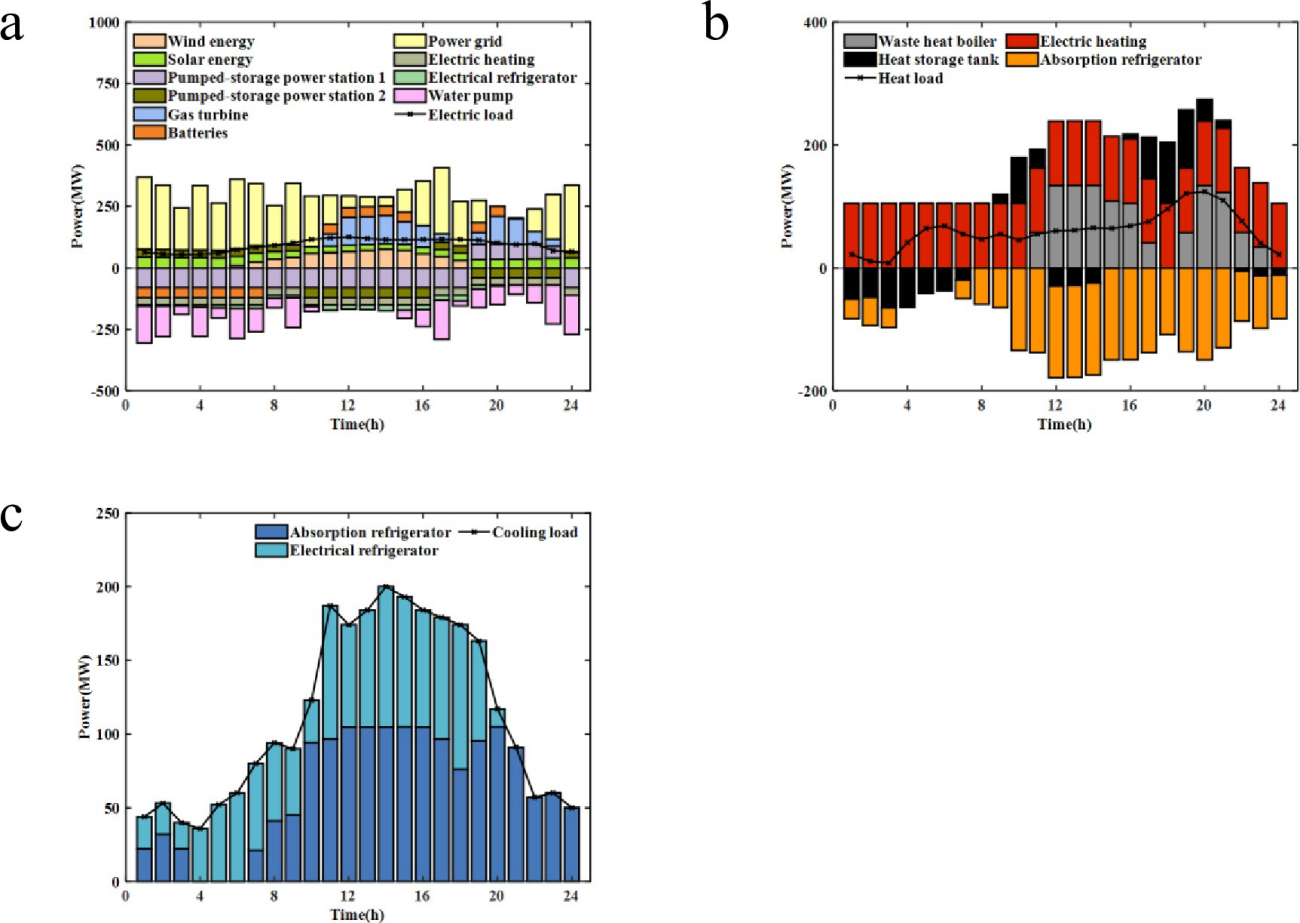
Balance of electrical/heat/cold load: (a) Balance of electrical load, (b) Balance of heat load, (c) Balance of cold load.

Fig 8A is an electrical load balance based on Case 4, where the power of batteries is positive for discharge, negative for charging, positive for power generation, and negative for pumped storage power. The results reveal that the electrical load is mainly borne by wind power, photovoltaic, large grid, and gas turbine; energy storage batteries and pumped storage power stations are mainly used for peak load shifting.

Fig 8B shows the heat balance based on Case 4, where the heat output of the waste heat boiler is positively correlated with the power of the gas turbine. Heat storage tank positively represents heat storage, which negatively represents heat release. The results show that the waste heat boiler and electric heating bear the main heat load in the whole period, and the waste heat boiler bear nearly half of the heat load, thus indicating that the introduction of the waste heat boiler can improve the heating capacity of the system. The heat storage tank undertakes the main task of peak regulation.

Fig 8C is the cold balance based on Case 4, and the electrical load is borne by an electric refrigerator and an absorption refrigerator. The results reveal that the absorption refrigerator bears the main cooling load in the whole period. This outcome indicates that the introduction of absorption refrigerator can transform the rich heat energy into cold energy and then improve the cooling capacity of the system, which has high economic value.

#### 4.2.4 Economic analysis

The cost of the objective function in Cases 1–4 is shown in Table 2. In addition, micro-source equipment operating costs are shown in Table 3A, and micro-source equipment maintenance costs are presented in Table 3B. Compared with Cases 1–3, the total cost and operating cost of Case 4 are all the best, and the maintenance cost is the second best. It verifies the superiority of economic dispatch considering the joint dispatch between pumped storage power stations 1 and 2 and water optimal dispatch 2 at the same time.

**Table 2:** Cost of the objective function in Cases 1–4 ($).

Case	Total cost	Operating cost	Maintenance cost	Environmental cost
1	671229.22	624710.94	32449.51	14068.77
2	567149.24	521968.60	32028.46	13152.18
3	496094.56	449513.10	32411.33	14170.13
4	459787.27	413767.41	32256.62	13763.24

**Table 3:** Micro-source equipment operating/maintenance costs ($).

**(a) Micro-source equipment operating costs ($)**					
Case	Gas turbine cost	Power grid purchase cost	Operation cost of seawater desalination	Penalty cost of scenery abandonment	Start and stop cost of the pumped storage unit
1	93021.61	388739.22	110476.73	32473.38	0
2	86094.55	304729.40	110476.73	18729.82	1938.10
3	92874.39	244238.99	110476.73	1624.79	298.20
4	90284.34	211664.54	110476.73	0	1341.80
**(b) Micro-source equipment maintenance costs($)**					
Facility	Case 1	Case 2	Case 3	Case 4	
Gas turbine	3206.28	2997.39	3229.38	3136.65	
Photovoltaic cells	1308.00	1308.00	1308.00	1308.00	
Wind turbine	3347.40	3347.40	3347.40	3347.40	
Batteries	8173.08	8176.00	8176.00	8176.00	
Electric heating	7537.48	7531.20	7531.20	7531.20	
Waste heat boiler	343.53	321.15	346.01	336.07	
Heat storage tank	2763.34	2741.64	2691.42	2710.02	
Electrical refrigerator	453.92	475.68	452.48	461.76	
Absorption refrigerator	5316.48	5130.00	5329.44	5249.52	

Compared with case 1, the total cost of Case 2 is reduced by $104,079.98 mainly because the introduction of pumped storage power station 1 in Case 2 reduces wind and solar energy curtailment. Thus, the penalty cost of scenery abandonment and the cost of power generation equipment are reduced. Among them, the penalty cost of scenery abandonment decreases by $13,743.56, the power purchase cost of power grid decreases by $84,009.82, and the cost of gas turbine decreases by $6,927.06.

Compared with Case 2, the total cost of Case 3 is reduced by $71,054.68 mainly because Case 3 considers water optimal dispatch 1. Therefore, pumped storage power station 1 is in the state of pumped storage for most of the time, which further reduces wind and solar energy curtailment. Among them, the penalty cost of scenery abandonment decreases by $17,105.03, the power purchase cost of power grid decreases by $60,490.42, and the cost of gas turbine increases by only $6,779.84.

For Case 4, the joint dispatch between pumped storage power stations 1 and 2 and water optimal dispatch 2 is considered. While further reducing the penalty cost of scenery abandonment, it also stores the surplus wind and solar power during the low load period and converts it into the scarce power during the peak load period. Thus, the total cost of the objective function in this case is the lowest. Compared with Case 1, the total cost is reduced by $211,441.95. Among them, operating, maintenance, and environmental costs decreases by $210,943.53, $192.89, and $305.53$, respectively.

## 5 Conclusion

To improve the economy of the integrated energy system and the capacity of accommodating wind and solar energy, this study takes into account the comprehensive flexible operation of pumped storage and establishes an IES optimal scheduling model and solves the model by improving the GWO. The conclusions obtained from the example analysis are as follows:

1. Considering the system water scheduling can reduce the impact of storage constraints on pumped storage power stations.
2. Considering the joint dispatch between pumped storage power stations 1 and 2 can make IES accommodate all the wind energy and solar energy.
3. At the same time, considering the joint dispatch between pumped storage power stations 1 and 2 and system water scheduling is economical, as the total cost of the economic dispatch of the integrated energy system is reduced by about 31.50%.
4. Considering the rich connotation of IES interconnection and interaction, the research of system planning can be further deepened from the aspects of multi-energy flow coupling and multi-system integration.

## Supporting information

S1 Appendix Mathematical models of each micro-source component in the system. (DOCX)

S2 Appendix The calculation formula of economic cost. (DOCX)

S3 Appendix The improved method and solution process of the algorithm. (DOCX)

S1 Table Pumped storage unit parameters. (DOCX)

S2 Table Reservoir parameters. (DOCX)

S3 Table Micro-source equipment correlation coefficient. (DOCX)

S4 Table Micro-source equipment maintenance price ($/MW·h). (DOCX)

## Abbreviations

Acronyms
IES: Integrated Energy System
WSPS: Wind-solar-pump storage energy supply system (WSPS)
water optimal dispatch 1: The water scheduling between the reservoir of seawater desalination plant and reservoirs of pumped storage power station 1
water optimal dispatch 2: The water scheduling between the reservoir of seawater desalination plant and reservoirs of pumped storage power station 1 and 2
$P_{MT}^{e}$: The power generation of the gas turbine
$P_{MT}^{h}$: The waste heat power generated by the gas turbine
$P_{RB}^{h}$: The heat production power of the waste heat boiler
$P_{EB}^{e}$: The input electric power of electric heating
$P_{EB}^{h}$: The output heat power of electric heating
$E_{HST}^{h}$: The heat storage capacity of the heat storage tank
$P_{HST}^{h,c}/P_{HST}^{h,d}$: The heat storage or release of the heat storage tank
$P_{AC}^{h}$: The input thermal power of the absorption refrigerator
$P_{EC}^{e}$: The input electric power of electric refrigerator
$P_{W}^{e}$: The generate power of wind turbine
$P_{V}^{e}$: The electric generation power of micro turbine
$P_{PS}^{e}$: The current power of the pumped storage unit
$C_{DES}^{w}$: The water production of seawater desalination device
$P_{DES}^{e}$: The power consumption of desalinated seawater
$P_{ES}^{c}$/$P_{ES}^{d}$: The heat storage or release of the battery
