# Supplementary material for: Integrated energy system optimal scheduling considering the comprehensive and flexible operation mode of pumping storage
Source: PLoS One. 2022 Oct 5;17(10):e0275514. doi: 10.1371/journal.pone.0275514 (PMC9534450; doi:10.1371/journal.pone.0275514)
Supplement: S1 Appendix — (DOCX) [file pone.0275514.s001.docx]

## Gas turbine

The gas turbine consumes natural gas to provide stable electric power for the system and generates a certain amount of waste heat. The relevant formula is as follows:

|  | $\left\{\begin{matrix} P_{MT}^{e}\left(t\right)=P_{MT}^{gas}\left(t\right)\eta_{MT}^{e} \\ P_{MT}^{gas}\left(t\right)=k_{gas}Q_{MT}^{gas}\left(t\right) \\ P_{MT}^{h}\left(t\right)=P_{MT}^{e}\left(t\right)\frac{\left(1-\eta_{MT}^{e}-\eta_{MT}^{loss}\right)}{\eta_{MT}^{e}} \end{matrix}\right.$ | (1) |
| --- | --- | --- |

where, $\eta_{MT}^{e}$ is the power generation efficiency of the gas turbine; $k_{gas}$ is the calorific value of natural gas, which is generally taken as 9.7kW^.^h/m^3^; $Q_{MT}^{gas}\left(t\right)$ is the consumption of natural gas by the gas turbine in period $t$; $P_{MT}^{h}\left(t\right)$ is the waste heat power generated by the gas turbine in period $t$, which is mainly expressed as exhaust gas; $\eta_{MT}^{loss}$ is the heat loss efficiency of the gas turbine.

## Waste heat boiler

The waste heat boiler expends the unused heat of the gas turbine as the heat source to provide users with available heat power, thereby improving the energy utilization rate of the system and reducing the expenditure due to the heat load:

|  | $\left\{\begin{matrix} P_{RB}^{h}\left(t\right)=P_{MT}^{h}\left(t\right)\eta_{RB}^{h} \\ P_{RB}^{h,\text{min}}\leq P_{RB}^{h}\left(t\right)\leq P_{RB}^{h,\text{max}} \end{matrix}\right.$ | (2) |
| --- | --- | --- |

where, $\eta_{RB}^{h}$ is the thermal efficiency of the waste heat boiler; $P_{RB}^{h,\text{min}}$ and $P_{RB}^{h,\text{max}}$ are the upper and lower limits of the heat production power of the waste heat boiler, respectively.

## Electric heating

Electric heating can use the surplus electricity of IES to produce heat, which is the main stable heat source for system heating. The relevant formula is as follows:

|  | $\left\{\begin{matrix} P_{EB}^{h}\left(t\right)=P_{EB}^{e}\left(t\right)COP_{EB}^{h} \\ P_{EB}^{e,\text{min}}\leq P_{EB}^{e}\left(t\right)\leq P_{EB}^{e,\text{max}} \end{matrix}\right.$ | (3) |
| --- | --- | --- |

where, $COP_{EB}^{h}$ is the heating coefficient of electric heating; $P_{EB}^{h,\text{min}}$ and $P_{EB}^{h,\text{max}}$ are the upper and lower limits of the input electric power of electric heating respectively.

## Heat storage tank

The heat storage tank can save the surplus heat of IES for the next use and has the effect of smoothing the fluctuation of heat load. The relevant formula is as follows:

|  | $\left\{\begin{matrix} S_{HST}\left(t+1\right)=S_{HST}\left(t\right)+\eta_{HST}^{c}P_{HST}^{c}\left(t+1\right)-P_{HST}^{d}\left(t+1\right)/\eta_{HST}^{d} \\ S_{HST}^{c,\min}\leq S_{HST}\left(t\right)\leq S_{HST}^{c,\max} \\ I_{HST}^{c}\left(t\right)P_{HST}^{c,\min}\leq P_{HST}^{c}\left(t\right)\leq I_{HST}^{c}\left(t\right)P_{HST}^{c,\max} \\ I_{HST}^{d}\left(t\right)P_{HST}^{d,\min}\leq P_{HST}^{d}\left(t\right)\leq I_{HST}^{d}\left(t\right)P_{HST}^{d,\max} \\ I_{HST}^{c}/I_{HST}^{d}\in\left\{0,1\right\} \\ I_{HST}^{c}\left(t\right)+I_{HST}^{d}\left(t\right)\leq1 \end{matrix}\right.$ | (4) |
| --- | --- | --- |

where, $S_{HST}\left(t\right)$ is the heat storage capacity of the heat storage tank in period $t$; $S_{HST}^{c,\max}$/$S_{HST}^{c,\min}$are the upper and lower limits of the electric quantity of heat storage tank, respectively; $\eta_{HST}^{c}$/$\eta_{HST}^{d}$ are the charging/discharging efficiency of the heat storage tank, respectively. $P_{HST}^{c,\max}$/$P_{HST}^{c,\min}$ are the upper and lower limits of the charge power of the heat storage tank, respectively; $P_{HST}^{d,\max}$/$P_{HST}^{d,\min}$ are the upper and lower limits of the discharge power of the heat storage tank, respectively; $I_{HST}^{c}$/$I_{HST}^{d}$ is the status flag bit of the heat storage tank in period t.

## Absorption refrigerator

An absorption refrigerator can use the excess heat of IES for refrigeration, which can reduce the use of electrical energy in the system and improve the energy efficiency of the system. The relevant formula is as follows:

|  | $\left\{\begin{matrix} P_{AC}^{c}\left(t\right)=P_{AC}^{h}\left(t\right)COP_{AC} \\ P_{AC}^{h,\text{min}}\leq P_{AC}^{h}\left(t\right)\leq P_{AC}^{h,\text{max}} \end{matrix}\right.$ | (5) |
| --- | --- | --- |

where, $COP_{AC}$ is the refrigeration coefficient of the absorption chiller; $P_{AC}^{h,\text{max}}$ are the upper and lower limits of the input thermal power of the absorption refrigerator, respectively.

## Electrical refrigerator

Electric refrigerator can use the surplus power of IES for refrigeration and is the main stable cold source of the system. The relevant formula is as follows:

|  | $\left\{\begin{matrix} P_{EC}^{c}\left(t\right)=P_{EC}^{e}\left(t\right)COP_{EC} \\ P_{EC}^{e,\text{min}}\leq P_{EC}^{e}\left(t\right)\leq P_{EC}^{e,\text{max}} \end{matrix}\right.$ | (6) |
| --- | --- | --- |

where, $\eta_{EC}^{c}$ is the refrigeration coefficient of electric refrigerator; $P_{EC}^{e,\text{min}}$ and $P_{EC}^{c,\text{max}}$ are the upper and lower limits of the input electric power of the electric refrigerator, respectively.

## Wind turbine

The power generated by wind turbines is highly susceptible to wind speed. The specific relationship is as follows:

|  | $P_{W}^{e}=\left\{\begin{matrix} 0 & V\left\langle V_{in}orV\right\rangle V_{out} \\ \frac{P_{W,r}^{e}\left(V-V_{in}\right)}{\left(V_{r}-V_{in}\right)} & V_{in}<V<V_{r} \\ P_{W,r}^{e} & V_{r}<V<V_{out} \end{matrix}\right.$ | (7) |
| --- | --- | --- |

where, $P_{W}^{e}$ are the rated power of the wind turbine; $V$, $V_{in}$, $V_{r}$, and $V_{out}$ are the real-time wind speed, cut-in wind speed, rated wind speed, and cut-out wind speed of the wind turbine, respectively.

## Photovoltaic cells

The power generation of photovoltaic cells is affected by environmental factors and usually fluctuates greatly. The specific relationship is as follows:

|  | $\left\{\begin{matrix} P_{V}^{e}=P_{V,s}^{e}\eta_{v}^{e} \\ \eta_{v}^{e}=\frac{G}{G_{s}}\left(1+k\left(\begin{matrix} T_{e}+ \\ \frac{G}{800}\left(T_{n}-20\right)-T_{s} \end{matrix}\right)\right) \end{matrix}\right.$ | (8) |
| --- | --- | --- |

where, $P_{V,s}^{e}$, $G_{s}$, and $T_{s}$ are the reference output power, light intensity, and surface temperature of photovoltaic cells under standard conditions, respectively; $\eta_{v}^{e}$ is the actual power output coefficient of the photovoltaic cells; G is the actual light intensity; $k$ is the power stability factor; $T_{e}$ and $T_{s}$ are the ambient temperature and the component temperature, respectively.

## Pumped storage unit

The pumped storage power station is mainly used to absorb the electric energy generated by photovoltaic cells and wind energy to reduce the impact of wind and wind energy on the grid. The relevant formula is as follows:

|  | $\left\{\begin{matrix} U_{ps}^{d}\left(t\right)=U_{ps}^{d}\left(t-1\right)+m_{a1}\left(t\right)-m_{b1}\left(t\right)+m_{2a}\left(t\right) \\ U_{ps}^{u}\left(t\right)=U_{ps}^{u}\left(t-1\right)-m_{a1}\left(t\right)+m_{b1}\left(t\right)-m_{3a}\left(t\right) \end{matrix}\right.$ | (9) |
| --- | --- | --- |

where, $U_{ps}^{u}\left(t\right)$ and $U_{ps}^{d}\left(t\right)$ are the water storage capacity of the high/low reservoir during period $t$, respectively.

|  | $\left\{\begin{matrix} m_{a_{1}}\left(t\right)=\sum_{k=1}^{N_{1}} m_{k}^{d}\left(t\right) \\ m_{b1}\left(t\right)=\sum_{k=1}^{N_{1}} m_{k}^{c}\left(t\right) \\ P_{ps1}^{d}\left(t\right)=\sum_{k=1}^{N_{1}} P_{ps1,k}^{d}\left(t\right) \\ P_{ps1}^{c}\left(t\right)=\sum_{k=1}^{N_{1}} P_{ps1,k}^{c}\left(t\right) \end{matrix}\right.$ | (10) |
| --- | --- | --- |

where, $N_{1}$ is the number of units configured in pumped storage station 1; $m_{k}^{d}\left(t\right)$ is the water consumption required for the discharge of the pumped storage unit k in the period $t$; $m_{k}^{c}\left(t\right)$ is the pumping amount of the pumped storage unit k in period $t$; $P_{ps1,k}^{c}\left(t\right)$/$P_{ps1,k}^{d}\left(t\right)$ are the charging/discharging power of the pumped-storage unit k in period $t$, respectively.

|  |  | (11) |
| --- | --- | --- |

where, $U_{ps}^{u,\text{min}}\left(t\right)$, $U_{ps}^{u,\text{max}}\left(t\right)$,$U_{ps}^{d,\text{min}}\left(t\right)$, and $U_{ps}^{d,\text{max}}\left(t\right)$ are the upper and lower limits of the storage capacity of the high/low reservoirs during period $t$, respectively; $U_{ps}^{u}\left(start\right)$ and $U_{ps}^{u}\left(end\right)$ are the beginning/end storage capacity of the high/low reservoir, respectively; and are the upper and lower limits of the difference between the beginning and end storage capacity, respectively.

## Seawater desalination device

Seawater desalination system is usually composed of multiple seawater desalination units and reservoirs. Reverse osmosis (RO) process is widely used because of its low unit energy consumption and high degree of equipment modularization. Therefore, the seawater desalination unit accommodates reverse osmosis process in this research. The relevant formula is as follows:

|  |  | (12) |
| --- | --- | --- |

where, $P_{DES}^{e,w}\left(t\right)$ is the power consumption of desalinated seawater by the seawater desalination device in period $t$; $\eta_{DES}^{w}$ and are the water yield per unit power and the pumping power per unit water of the seawater desalination unit, respectively.

## Batteries

The battery can save the surplus power of IES for next use, which can stabilize the fluctuation of the electric load. The relevant formula is as follows:

|  | $\left\{\begin{matrix} S_{EB}\left(t+1\right)=S_{EB}\left(t\right)+\eta_{EB}^{c}P_{EB}^{c}\left(t+1\right)-P_{EB}^{d}\left(t+1\right)/\eta_{EB}^{d} \\ S_{EB}^{c,\min}\leq S_{EB}\left(t\right)\leq S_{EB}^{c,\max} \\ I_{EB}^{c}\left(t\right)P_{EB}^{c,\min}\leq P_{EB}^{c}\left(t\right)\leq I_{EB}^{c}\left(t\right)P_{EB}^{c,\max} \\ I_{EB}^{d}\left(t\right)P_{EB}^{d,\min}\leq P_{EB}^{d}\left(t\right)\leq I_{EB}^{d}\left(t\right)P_{EB}^{d,\max} \\ I_{EB}^{c}/I_{EB}^{d}\in\left\{0,1\right\} \\ I_{EB}^{c}\left(t\right)+I_{EB}^{d}\left(t\right)\leq1 \end{matrix}\right.$ | (13) |
| --- | --- | --- |

where, $S\left(t\right)$ is the electric quantity of battery; $S_{EB}^{c,\max}$/$S_{EB}^{c,\min}$are the upper and lower limits of the electric quantity of battery, respectively; $\eta_{ES}^{c}$/$\eta_{ES}^{d}$ are the charging/discharging efficiency of the battery, respectively; $P_{EB}^{c,\max}$/$P_{EB}^{c,\min}$ are the upper and lower limits of the charge power of the battery, respectively; $P_{EB}^{d,\max}$/$P_{EB}^{d,\min}$ are the upper and lower limits of the discharge power of the battery, respectively; $I_{EB}^{c}$/$I_{EB}^{d}$ is the status flag bit of the battery in period t.
