# Supplementary material for: Integrated energy system optimal scheduling considering the comprehensive and flexible operation mode of pumping storage
Source: PLoS One. 2022 Oct 5;17(10):e0275514. doi: 10.1371/journal.pone.0275514 (PMC9534450; doi:10.1371/journal.pone.0275514)
Supplement: S2 Appendix — (DOCX) [file pone.0275514.s002.docx]

Total cost objective function:

|  | $F=\text{min}\left(F_{x}+F_{y}+F_{z}\right)$ | (1) |
| --- | --- | --- |

Operating cost objective function:

|  | $\left\{\begin{matrix} F_{x}=F_{MT}+F_{PS,\text{se}}+F_{WV,dis}+F_{DES,w}+F_{grid} \\ F_{MT}=\sum_{t\in\text{ }\Omega_{MT}} MT_{gas}\left(t\right)\xi_{gas} \\ MT_{gas}\left(t\right)=aP_{MT}^{e}\left(t\right)^{2}+bP_{MT}^{e}\left(t\right)+c \\ F_{PS,\text{se}}=F_{PS,p}+F_{PS,e} \\ m_{i}^{0},n_{i}^{0}=0 \\ F_{PS,p}=\sum_{t\in\Omega_{PS}} \left(I_{psi}^{c}\left(t\right)-I_{psi}^{c}\left(t-1\right)\right)\xi_{PS,p} \\ F_{PS,e}=\sum_{t\in\Omega_{PS}} \left(I_{psi}^{d}\left(t\right)-I_{psi}^{d}\left(t-1\right)\right)\xi_{PS,e} \\ F_{WV,dis}=\sum_{t\in\Omega_{WV}} P_{WV,dis}\left(t\right)\xi_{WV,dis} \\ F_{DES,w}=\sum_{t\in\Omega_{DES}} m_{1}\left(t\right)\xi_{DES,w} \\ F_{grid}=\sum_{t\in\text{ }\Omega_{E}} \left(P_{\text{grid}}^{e}\left(t\right)\xi_{grid}\left(t\right)\right) \end{matrix}\right.$ | (2) |
| --- | --- | --- |

Maintenance cost objective function:

|  | $F_{y}=\sum_{t\in\Omega_{y}} \sum_{i\in\Omega_{yi}} P_{i}\left(t\right)\theta_{i}$ | (3) |
| --- | --- | --- |

Environmental cost objective function:

|  | $F_{z}=\sum_{t\in\Omega_{MT}} P_{MT}^{e}\left(t\right)\rho_{c}\xi_{c}$ | (4) |
| --- | --- | --- |

where, $\Omega_{MT}$, $\Omega_{PS}$, $\Omega_{WV}$, $\Omega_{DES}$, $\Omega_{E}$, $\Omega_{y}$, and $\Omega_{yi}$ are the set of gas turbine output period, pumped storage output period, wind output period, seawater desalination unit output period, power purchase period, equipment maintenance period, and equipment maintenance period, respectively; $F_{MT}$, $F_{PS,se}$, $F_{WV,dis}$, $F_{DES,w}$, and $F_{grid}$ are the natural gas cost of the gas turbine, the start and stop cost of the pumped storage unit, the penalty cost of wind and solar energy curtailment, the operation cost of seawater desalination, and the power purchase cost of the power grid, respectively; $\xi_{gas}$, $\xi_{PS,p}$, $\xi_{PS,e}$, $\xi_{DES,w}$, $\xi_{WV,dis}$, and $\xi_{grid}$ are the unit price of gas turbine fuel, the single pumping start-up cost of a single pumped storage unit and the single power generation start-up cost, the operating cost coefficient of seawater desalination, and the penalty cost of scenery abandonment, respectively. The unit price of electricity purchased by the power grid. $MT_{gas}\left(t\right)$ is the amount of fuel required for a gas turbine in $t$ period, where $a$, $b$, and $c$ are the proportional factors; $P_{i}\left(t\right)$ is the working power of device $i$ in $t$ period; $\theta_{i}$ is the maintenance unit price of device $i$; $\rho_{c}$ is the intensity of carbon dioxide emission per unit power of the gas turbine; $P_{i}$ is the total power generated by the fossil fuel unit.
