# Supplementary material for: Integrated energy system optimal scheduling considering the comprehensive and flexible operation mode of pumping storage
Source: PLoS One. 2022 Oct 5;17(10):e0275514. doi: 10.1371/journal.pone.0275514 (PMC9534450; doi:10.1371/journal.pone.0275514)
Supplement: S3 Appendix — (DOCX) [file pone.0275514.s003.docx]

The gray wolf optimizer is similar to particle swarm optimization algorithm in that it requires a better initial solution set. The selection of the solution set affects the global search ability and local convergence ability of the whole algorithm. At the beginning of optimization, Monte Carlo was used to simulate the continuous hunting process of the gray wolf population. Better individuals were selected to form the initial population during the hunting process to ensure the global population and obtain a reasonable initial solution.

In addition, the original gray wolf algorithm prevents the algorithm from falling into local optimum by selecting three leading wolves $\alpha$,$\beta$, and $\gamma$. However, when all three leading wolves are local optimum solutions, the algorithm still has the risk of falling into the local optimum. This algorithm divides the initial wolf groups by FCM clustering algorithm. By comparing the individual fitness of each group, the leading wolf $\alpha$ is selected as the representative of each group, which increases the diversity of the population and prevents the algorithm from falling into the local optimum. Then, the position of each group of wolf $\alpha$ is updated by formula (1) (displacement update formula of particle swarm algorithm). In this scenario, each group of wolf $\alpha$ is the best in a small range around it, which avoids the risk in which the search results of each group will fall into local suboptimal when the individual search range of wolf is wide and the leading wolf $\alpha$ is local suboptimal.

|  | $\left\{\begin{matrix} \Delta w=\frac{\left(w_{\text{max}}-w_{\text{min}}\right)\left[f\left(x_{i}^{d}\right)-f_{\text{min}}^{d}\right]}{f_{average}^{d}-f_{\text{min}}^{d}} \\ w_{i}^{d}=\left\{\begin{matrix} w_{\text{min}}+\Delta w & ,f\left(x_{i}^{d}\right)\leq f_{average}^{d} \\ w_{\text{max}} & ,f\left(x_{i}^{d}\right)>f_{average}^{d} \end{matrix}\right. \\ V_{i}^{k+1}=w_{i}^{d}V_{i}^{k}+c_{1}rand_{1}\left(P_{best}-X_{i}^{k}\right)+ \\ c_{2}rand_{2}\left(G_{best}-X_{i}^{k}\right) \\ X_{i}^{k+1}=X_{i}^{k}+V_{i}^{k+1} \end{matrix}\right.$ | (1) |
| --- | --- | --- |

where, $w_{i}^{d}$ is the inertia weight; $w_{\text{min}}$ and $w_{\text{max}}$ are the preset minimum and maximum inertia coefficients, respectively. Generally, $w_{\text{min}}$ is 0.4, and $w_{\text{max}}$ is 0.9; $f_{average}^{d}$ is the average fitness of all particles at iteration d; $f_{\text{min}}^{d}$ is the average fitness of all particles at iteration d; $V_{i}^{k}$ is the k-th search speed and direction of the i-th particle; $X_{i}^{k}$ is the location of the k-th search for the i-th particle; $P_{best}$ is an individual optimal solution; $G_{best}$ is the optimal solution for the population; $c_{1}$ and $c_{2}$ are learning factors that enable particles to self-summarize and learn from excellent individuals in the group, respectively; $rand_{1}$ and $rand_{2}$ are uniformly distributed random number between 0 and 1, respectively. At the same time, equation (2) (Detection formula of Harris Hawk algorithm) continuously updates the escape location of the prey and uses Monte Carlo stochastic simulation to find the best escape location for the prey, thus avoiding prematurely jumping out of the region when the location of the prey is local suboptimal in a small surrounding area, thereby reducing the probability that the optimal result is local optimal.

|  | $\left\{\begin{matrix} \Delta X_{1}=-2r_{2}X\left(t\right) \\ \Delta X_{2}=X_{rabbit}\left(t\right)-X_{m}\left(t\right) \\ A=lb+r_{4}\left(ub-lb\right) \\ X\left(t+1\right)=\left\{\begin{matrix} X_{rand}\left(t\right)-r_{1}\left\vert\Delta X_{1}\right\vert& ,q\geq0.5 \\ \Delta X_{2}-r_{3}A & ,q<0.5 \end{matrix}\right. \\ X_{m}\left(t\right)=\sum_{k=1}^{M} \frac{X_{k}\left(t\right)}{M} \end{matrix}\right.$ | (2) |
| --- | --- | --- |

where, $X\left(t\right)$ and $X\left(t+1\right)$ are the position of the individual in the current and next iteration, respectively; $t$ is the number of iterations; $X_{rand}\left(t\right)$ is the location of an individual randomly selected; $X_{rabbit}\left(t\right)$ is the position of the prey, that is, the position of the individual with the best fitness; $r_{1}$, $r_{2}$, $r_{3}$, and $r_{4}$ are random numbers between [0,1]; $q$ is used to select the strategy to be accommodated randomly; $X_{k}\left(t\right)$ is the position of the k-th individual in the population; $M$is the population size.

The remaining wolves except the leading wolf $\alpha$ are randomly sampled with probability p to select the pursuer, and their positions are updated with formula (3) (displacement update formula of the gray wolf optimizer), which ensures the searching ability of the pursuer within the group. The introduction (4) (velocity update formula of particle swarm algorithm) is updated twice, and formula (4) introduces the current global optimal solution as reference. It improves the hunting ability of each group of wolf hunters and reduces the probability of wolves falling into local optimum. The remaining wolves except the leading wolf $\alpha$ were randomly sampled with probability (1-p) to select the watchers, and their positions were updated with formula (4) (the displacement update formula of the bat algorithm), which reduces the probability of the prey escaping and improves the ability of the algorithm to jump out of the local area.

|  | $\left\{\begin{matrix} D=\left\vert C\cdot X_{p}\left(t\right)-X\left(t\right)\right\vert\\ X\left(t+1\right)=X_{p}\left(t\right)-A\cdot D \\ A=2a\cdot r_{1}-a \\ C=2\cdot r_{2} \end{matrix}\right.$ | (3) |
| --- | --- | --- |

where, $t$ is the current number of iterations; $D$ is the distance between an individual and his prey; $A$ and $C$ are coefficients vectors; $X_{p}\left(t\right)$ represents the location vector of the prey; $X\left(t\right)$ represents the position vector of the current wolf; $a$ decreases linearly from 2 to 0 throughout the iteration; $r_{1}$ and $r_{2}$ are random vectors in [0,1].

|  | $\left\{\begin{matrix} f_{i}=f_{\text{min}}+\left(f_{\text{max}}-f_{\text{min}}\right)\beta\\ v_{i}^{t}=v_{i}^{t-1}+\left(x_{i}^{t}-x_{\text{*}}\right)f_{i} \\ x_{i}^{t}=x_{i}^{t-1}+v_{i}^{t} \end{matrix}\right.$ | (4) |
| --- | --- | --- |

where, $\beta$ is random vectors in [0,1]; $x_{\text{*}}$ is the current optimal individual location; $f_{i}$ is the frequency of sound waves emitted by bats with a value between [$f_{\text{min}}$,$f_{\text{max}}$].

Finally, a memory bank is added to save the position and other parameters of each group of wolves during the first n iterations, and the crowded individuals are selected as the current individuals through crowding analysis, which ensures the diversity of each group of populations and avoids wolves. The pack is overcrowded so that the wolf pack falls into a local optimum and loses its search ability at the end of the iteration.
