# Supplementary material for: Integrated energy system optimal scheduling considering the comprehensive and flexible operation mode of pumping storage
Source: PLoS One. 2022 Oct 5;17(10):e0275514. doi: 10.1371/journal.pone.0275514 (PMC9534450; doi:10.1371/journal.pone.0275514)
Supplement: S1 Table — (DOCX) [file pone.0275514.s004.docx]

| Pumped storage unit | Power Generation State Startup Cost /$ | Starting cost of pumping status /$ | Pumping power /MW | Pumping flow /(m^3^/h) | Power Segment Number | Segment Start Power /MW | End power /MW | Micro-increase water consumption /(m^3^/MW·h) |
| --- | --- | --- | --- | --- | --- | --- | --- | --- |
| PU-1 | 111.8 | 149.1 | 40.1 | 1500 | 1 | 10 | 18 | 45.0 |
|  |  |  |  |  | 2 | 18 | 24 | 50.0 |
|  |  |  |  |  | 3 | 24 | 30 | 56.7 |
| PU-2 | 111.8 | 149.1 | 40.1 | 1500 | 1 | 10 | 20 | 18.0 |
|  |  |  |  |  | 2 | 20 | 50 | 20.0 |
|  |  |  |  |  | 3 | 50 | 60 | 22.0 |
