# Supplementary material for: Integrated energy system optimal scheduling considering the comprehensive and flexible operation mode of pumping storage
Source: PLoS One. 2022 Oct 5;17(10):e0275514. doi: 10.1371/journal.pone.0275514 (PMC9534450; doi:10.1371/journal.pone.0275514)
Supplement: S2 Table — (DOCX) [file pone.0275514.s005.docx]

| Reservoir | High Reservoir /(10^3^m^3^) | | | Low Reservoir /(10^3^m^3^) | | |
| --- | --- | --- | --- | --- | --- | --- |
|  | Lower capacity limit | Upper capacity limit | Initial water volume | Lower capacity limit | Upper capacity limit | Initial water volume |
| U-1 | 5 | 15 | 10 | 35 | 45 | 40 |
| U-2 | 2 | 8 | 5 | 17 | 23 | 20 |
