# Supplementary material for: Integrated energy system optimal scheduling considering the comprehensive and flexible operation mode of pumping storage
Source: PLoS One. 2022 Oct 5;17(10):e0275514. doi: 10.1371/journal.pone.0275514 (PMC9534450; doi:10.1371/journal.pone.0275514)
Supplement: S3 Table — (DOCX) [file pone.0275514.s006.docx]

| Facility | $\eta_{MT}^{e}/\eta_{MT}^{loss}$ | $\eta_{EB}^{h}$/$COP_{EB}$ | $COP_{EC}$ | $COP_{AC}$ | $\eta_{ES}^{c}$/$\eta_{ES}^{d}$ | $\eta_{HST}^{c}$/$\eta_{HST}^{d}$ |
| --- | --- | --- | --- | --- | --- | --- |
| Value | 0.35/0.1 | 0.875/4 | 4 | 0.7 | 0.95/0.95 | 0.92/0.92 |
