# Supplementary material for: Integrated energy system optimal scheduling considering the comprehensive and flexible operation mode of pumping storage
Source: PLoS One. 2022 Oct 5;17(10):e0275514. doi: 10.1371/journal.pone.0275514 (PMC9534450; doi:10.1371/journal.pone.0275514)
Supplement: S4 Table — (DOCX) [file pone.0275514.s007.docx]

| Facility | Gas turbine | Photovoltaic cells | Wind turbine | Batteries | Waste heat boiler | Electric heating | Heat storage tank | Absorption refrigerator | Electrical refrigerator |
| --- | --- | --- | --- | --- | --- | --- | --- | --- | --- |
| Price | 3.3 | 2 | 4.2 | 14.6 | 0.3 | 10.46 | 3.1 | 1.6 | 2.4 |
